# In-line warming reduces in-line pressure of subcutaneous infusion of concentrated immunoglobulins

**DOI:** 10.1007/s13346-023-01321-y

**Published:** 2023-03-15

**Authors:** Peter Leidenmühler, Joris Höfinghoff, Norbert Haider, Gerald Brachtl, Markus Weiller, Ivan Bilic, Bagirath Gangadharan

**Affiliations:** 1 Takeda Company, Baxalta Innovations GmbH, Donau City Strasse 7, Vienna, 1220 Austria; 2 Baxter AG, Takeda Company, Vienna, Austria

**Keywords:** Immunoglobulins, Subcutaneous administration, CUVITRU, rHuPH20, TAK-881, SCIG, SCIG 20%, fSCIG 20%, HYQVIA

## Abstract

Immunoglobulin replacement therapy is a life-saving treatment in patients with immunodeficiency and effective in the management of autoimmune disorders. Immunoglobulins are administered intravenously or subcutaneously, with the latter route reducing systemic reactions and providing an option for self-infusion, increasing patient convenience, while decreasing patient burden, healthcare utilization, and costs. A major limitation with subcutaneous administrations is the frequency of infusion due to limited volumes administrable into subcutaneous space, necessitating increased drug concentration, absorption, and dispersion. Increasing the concentration of immunoglobulins from 10 to 20% halves the required volume, but leads to higher dynamic viscosity, limiting infusion rate. Recombinant human hyaluronidase increases dispersion and absorption of immunoglobulins allowing administration of ≤ 600 mL per site, but does not change viscosity. Since the viscosity of fluids depends on temperature, we tested the feasibility of in-line warming of immunoglobulin formulations to physiological temperatures. In vitro analysis showed no negative impact of in-line warming to 38 °C on product quality. Subcutaneous infusion studies in pigs confirmed the feasibility of infusion rates of up to 7.5 mL/min with in-line warmed TAK-881, an immunoglobulin 20% facilitated with recombinant human hyaluronidase. In-line pressures were reduced compared with conventional immunoglobulin 20%, and local tolerance was not altered. Reduction of in-line pressures was more pronounced with thinner needle sets, indicating a potential benefit for patients. In summary, an in in-line warming device can circumvent the limitation of high viscosity, while product quality and local tolerance are maintained. The results of the presented studies warrant further testing in a phase 1 clinical study.

**Graphical Abstract:**

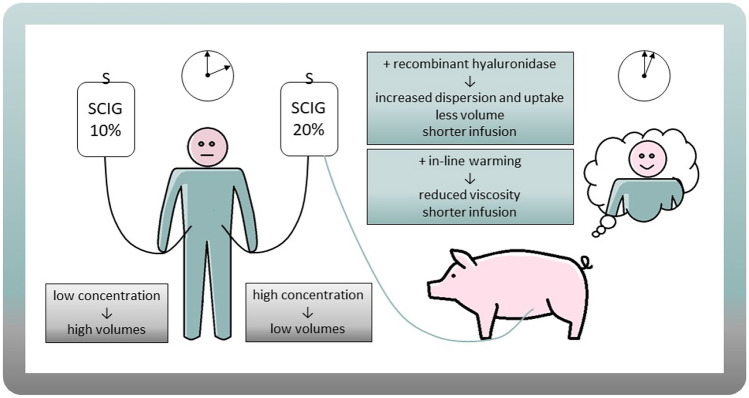

**Supplementary Information:**

The online version contains supplementary material available at 10.1007/s13346-023-01321-y.

## Introduction

Primary immunodeficiency disease (PIDD) is a class of disorders characterized by defects in both, humoral and cell-mediated immunity, resulting in increased susceptibility to infection, such as recurrent pyogenic infections and opportunistic infections [1, 2]. Common variable immunodeficiency (CVID) is the most prevalent form of PIDD and requires lifelong replacement therapy with immunoglobulin G (IgG) products, generally in the range of 0.3–0.6 g/kg body weight (BW) every 3–4 weeks [3, 4]. Similar doses are recommended for IgG replacement therapy in secondary immunodeficiency caused by malignancies like myeloma or chronic lymphocytic leukemia, resulting from acquired immune deficiency syndrome, or autologous hematopoietic stem cell transplantation [5–8].

Immunoglobulins are also effectively used in the treatment of autoimmune disorders, such as idiopathic thrombocytopenic purpura (ITP) [9], Kawasaki syndrome [10], and chronic inflammatory demyelinating polyradiculoneuropathy [11, 12]. The treatment of autoimmune disorders requires a higher dose of 2 g/kg/month IgG [13]. This dose is usually divided into separate doses of 1 g/kg BW over 2 days or 0.4 g/kg BW over 5 days.

Administration of high-dose IgG treatment regimens can be carried out by the intravenous (IV) route. However, due to the risk of severe adverse systemic reactions and the necessity for venous access, IV administration of immunoglobulin (intravenous immunoglobulin; IVIG) is commonly performed under the supervision of a medical professional and may require premedication with corticosteroids or antihistamines [14–16]. Subcutaneous (SC) administration of IgG (subcutaneous immunoglobulin; SCIG) is safe and equally efficacious to IVIG and was shown to result in more stable serum levels of IgG [17]. As SCIG rarely triggers adverse systemic reactions and does not require venous access, self-infusion at home is possible and can easily be mastered by the patient, including adolescent and the elderly [16–20].

Home treatment is associated with reduced costs [21] and is universally described to be appreciated by patients, who perceive more independence, less limitations in daily life, and a reduced sense of being sick or disabled [4, 20, 22–24]. This results in improved health-related quality of life and treatment satisfaction. Consequently, a number of studies have reported distinct preferences for SCIG in adult and pediatric patients with PIDD [4, 25, 26]. Furthermore, SCIG is an important alternative to IVIG treatment in patients with PIDD who cannot tolerate IV infusion due to a history of severe adverse drug reactions or comorbidities [14, 16, 17], and patients in whom stable venous access is difficult [27, 28]. In the latter individuals, SCIG prevents the need for surgically implanted devices such as indwelling catheters [27, 28].

A major disadvantage of SC therapy is the limited volume of administration at a single site (up to 60 mL) that is usually overcome by the use of multiple needle sites on a weekly or biweekly basis rather than a single IV infusion once every 3–4 weeks [14, 16, 17]. Furthermore, the bioavailability of IgG after SC administration is substantially lower (65–69%) compared to IV administration [29]. This may require an increased dose of IgG [30].

Multiple needle sticks and frequent administrations were reported to deter patients from treatment compliance, prompting some physicians to recommend against SCIG [16]. In addition to that, long infusion times for the volumes delivered with SCIG 10% are significantly related to negative patient experience and perspective, and a major barrier to patient adherence.

Two approaches to overcome this limitation can be pursued: first, increasing the concentration of the product, and second, increasing the volume of administration per site. Increasing the concentration of SCIG from 10 to 20% halves the required administered volume. However, a major challenge of increased concentration is the resulting higher viscosity of the solution, limiting infusion rate (e.g., 1 mL/min for CUVITRU™, a conventional SCIG 20% product), thus leading to longer infusion times. Furthermore, there are concerns regarding discomfort caused by the high administration volume per site, its potential effects on infusion site reactions [31].

Recombinant human hyaluronidase (rHuPH20) cleaves the repeating disaccharide subunits (N-acetyl-D-glucosamine and D-glucuronic acid) of hyaluronan, a polymeric, gel-like glycosaminoglycan (mucopolysaccharide) that limits the movement of fluids and other molecules in the subcutaneous tissue; rHuPH20 acts locally and transiently within the subcutaneous space to increase the dispersion and absorption of other, co-formulated or subsequently injected drugs and fluids. This localized effect results in a transient increase in permeability, allowing the IG component to disperse and to reach the systemic circulation more readily than without rHuPH20 [16]. The increased flow rates and increased delivery volumes compare favorably with SC administration without rHuPH20 [16]. Injectable hyaluronidase products have been in clinical use for over 60 years and are used to increase the tissue dispersion and absorption of other injected drugs [32].

Use of rHuPH20 allows administration of larger volumes of SCIG, has a potential to improve bioavailability of a 20% formulation compared to conventional SCIG, allowing the same dose volume as for IVIG, and thus enabling less frequent infusions.

Using SCIG 20% with a highly purified rHuPH20, also referred to as facilitated SCIG 20% (fSCIG 20%), potentially enables single-site SC administration of IgG with a lower volume than HYQVIA™, an SCIG 10% facilitated with rHuPH20, and at reduced infusion times compared to conventional SCIG 20%. Recombinant HuPH20 can increase dispersion and uptake of SCIG 20%, but cannot overcome the limitation of high viscosity.

Viscous forces are caused by molecules exerting attractive forces on each other. In liquids, dynamic viscosity is inversely proportional to temperature as increasing temperature causes an increase in the energy level of molecules in the liquid, leading to increased distance between molecules and decreased intermolecular attraction.

We hypothesized that warming a fSCIG 20% solution could circumvent the limitation of higher viscosity. The aim of the presented non-clinical in vitro and in vivo studies was to assess the feasibility of using an in-line warming device (set at 38–41 °C to target physiological temperatures at the needle site) to reduce viscosity of SCIG 20%, while ensuring product quality and local tolerance. Non-warmed SCIG and fSCIG 20% formulations were used as control articles and to assess the technical feasibility of SC infusion of these products at high flow rates, respectively.

## Methods

### Drug products

The following drug products were used: TAK-881, a fSCIG 20% containing 160 U/mL rHuPH20 in a separate vial, to be administered prior to infusion of SCIG 20% (Takeda, currently under development); HYQVIA™, a licensed fSCIG 10% containing 160 U/mL rHuPH20 in a separate vial, to be administered prior to infusion of SCIG 10% (Takeda); and CUVITRU™, a licensed SCIG 20% (Takeda).

### Determination of viscosity of immunoglobulins and influence on warming on product quality

The dynamic viscosity of immunoglobulins at concentrations from 100 to 200 mg/mL was determined at 20, 25, and 37 °C with a rolling-ball viscosimeter (Lovis 2000 ME, Anton Paar).

To assess the impact of in-line warming (38 °C) on the product quality of SCIG 20%, approximately 210 mL SCIG 20% was filled in a pooling bag, the line was attached to a peristaltic pump set to infusion rates of 2.5–7.5 mL/min (Infusomat Space, Braun), and thereafter passing by an infusion warmer set to 38 °C (BW 685 S, Biegler). After 200 mL SCIG 20% was collected on a balance with beaker (Schott), 10 mL aliquots were drawn and either analyzed immediately for micro-flow imaging (MFI Bot1, Proteinsimple), dynamic light scattering (Nano Ultra, Malvern), visual appearance, and turbidity (UV-1800, Shimadzu) or frozen at ≤  − 60 °C and analyzed for molecular size distribution via size exclusion-high performance liquid chromatography (SEC-HPLC; BIORAD Model AS-100 HRLC Automatic Sampling System, Waters 515 HPLC Pump, BIORAD Model 1790 Programmable UV/VIS Monitor).

### Determination of in-line pressures and local tolerability in pigs

For animal studies, all institutional and national guidelines for the care and use of laboratory animals were followed.

The animal studies used an adaption of a previously developed in vivo infusion model in pigs (Kang et al. [33]). Based on this previous experience, the pig model is considered the best suitable model to assess feasibility of SC administration of immunoglobulins [33]. A total of 30 pigs were anesthetized and placed in dorsal recumbency. Simultaneous SC infusions were administered in the left and right lower abdominal region. The test and control items, as well as infusion rates, are summarized in Table 1. Immunoglobulin infusions were attached to a 3-way stopcock, with or without a warming device (BW 685 S, Biegler, setpoint: 41 °C to target physiological temperatures at the needle site) in-between the infusion and the stopcock. At the other accessible side of the 3-way stopcock, a syringe with either rHuPH20 or buffer was attached. The line was attached to a pressure transducer (MLT0699, ADInstruments, Manufacturer), a temperature sensor (MLT415, ADInstruments) and administered to the animals using different needle sets (Venofix 19 gauge [G; Braun], Venofix 23 G [Braun], or BD-Saf T-intima 24 G [Becton Dickinson]; Table 1). The larger 19 G needle set was evaluated first to reduce the impact of the needle resistance on in-line pressure. Additional SC infusion experiments were performed using clinically relevant needle sizes of 23 G and 24 G.

**Table 1:** Summary of treatment in SC infusion studies for determination of in-line pressures and local tolerability in pigs

**Animals**	**Pre-treatment**	**Treatment**	**Needle size**	**Collateral pre-treatment**	**Collateral treatment**	**Collateral needle size**	**Flow rate (mL/min)**
*N* = 3	5 mL rHuPH20 (800 U)	50 mL warmed SCIG 20%	19 Ga	5 mL buffer	50 mL SCIG 20%	19 G	3
*N* = 3	5 mL rHuPH20 (800 U)	50 mL warmed SCIG 20%	19 G	5 mL buffer	50 mL SCIG 20%	19 G	4
*N* = 3	5 mL rHuPH20 (800 U)	50 mL warmed SCIG 20%	19 G	5 mL buffer	50 mL SCIG 20%	19 G	5
*N* = 3	5 mL rHuPH20 (800 U)	50 mL warmed SCIG 20%	19 G	5 mL buffer	50 mL SCIG 20%	19 G	7.5
*N* = 2	15 mL rHuPH20 (2400 U)	150 mL fSCIG 20%	19 G	15 mL rHuPH20 (1200 U)^d^	150 mL SCIG 10%	19 G	5
*N* = 2	5 mL rHuPH20 (800 U)	50 mL fSCIG 20%	19 G	5 ml rHUPh20 (400 U)^d^	50 mL fSCIG, 10%	19 G	5
*N* = 2	5 mL rHuPH20 (800 U)	50 mL warmed fSCIG 20%	23 Gb	5 mL buffer	50 mL SCIG 20%	23 G	5
*N* = 3	5 mL rHuPH20 (800 U)	50 mL SCIG 20%	24 G^c^	5 mL buffer	50 mL SCIG 20%	24 G	5
*N* = 3	5 mL rHuPH20 (800 U)	50 mL warmed SCIG 20%	24 G	5 mL buffer	50 mL SCIG 20%	24 G	5
*N* = 3	5 mL rHuPH20 (800 U)	50 mL SCIG 20%	24 G	5 mL rHuPH20 (800 U)	50 mL SCIG 20%	19 G	3
*N* = 3	16 mL rHuPH20 (2560 U)	160 mL warmed SCIG 20%	24 G	16 mL rHuPH20 (2560 U)	160 mL SCIG 20%	24 G	0.5, 1, 2, 3, 5^e^

*G* gauge, *SCIG* subcutaneous immunoglobulin, *N* number, *rHuPH20* recombinant human hyaluronidase PH20

^a^Venofix 19 G

^b^Venofix 23 G

^c^BD-Saf T-intima

^d^1:1 dilution for same volume as pre-treatment

^e^Flow rate ramp up: flow rate was increased every 10 min

In-line pressure was assessed during the entire infusion period. Mean and maximum in-line pressure during immunoglobulin infusion were determined and compared between treatments using an unpaired *T*-test (GraphPad Prism, version 8.0.2). For all infusions with warming, the temperature of the infusion was measured in-line. Results are presented in mean ± standard deviation (SD) or mean ± standard error of the mean (SEM).

To assess local tolerance, the following examinations were performed prior to the infusion, immediately at the end of the infusion, and at approximately 2, 4, and 24 h after completion of infusion: general welfare was monitored (appetite, animal behavior, and local reaction on the injection sites), quantitative evaluation of the injection sites was carried out (measuring the maximum length, width, and height of the bleb), post-treatment local infusion site assessment (qualitative scoring of erythema, swelling size, and bleb firmness using 4-point scoring system based on the 1992 OECD guidelines for grading skin reactions [34], a modified Draize test, calculating the sum of the scores for the 3 parameters), and histopathological examination of the infusion sites.

To mirror a potential clinical dosing regimen, an in vivo ramp up study with a high volume of 160 mL SCIG 20% using BD-Saf T-intima 24 G infusion set was performed. Subcutaneous infusion of warmed fSCIG 20% was compared to non-warmed fSCIG 20% at a flow rate of 0.5, 1, 2, 3, and 5 mL/min, with the flow rate being increased every 10 min.

### Pharmacokinetic study in pigs

The pharmacokinetics (PK) of warmed fSCIG 20%, conventional SCIG 20%, and in-line warmed SCIG 20% was compared after a single SC infusion in pigs. Three male pigs per group received either a SC dose of 400 mg/kg TAK-881 (flow rate: 5 mL/min), a SC dose of 400 mg/kg SCIG 20% (flow rate: 1 mL/min), or a SC dose of 400 mg/kg in-line warmed SCIG 20% (flow rate: 5 mL/min).

Whole blood samples were collected over 28 days at 0.5, 1, 3, 6, 12, 24, 48, 72, 96, 120, 168, 216, 288, 336, 408, 480, 576, and 672 h post-infusion. Serum was prepared for analysis of human IgG, which was analyzed using a sandwich enzyme-linked immunosorbent assay (ELISA) for total human IgG concentrations (coating antibody: mouse anti-human Ig lambda light chain [bound + free] 2G9, BioRad; detection antibody: goat anti-human Ig (H + L) HRP; Abcam; Maxisorb plates, Nunc, Plate reader: Glomax [Promega]). Pharmacokinetic parameters were determined based upon individual PK profiles using a non-compartmental model with WinNonlin^®^ (version 6.4). Results are presented in mean ± SD. PK values were compared between treatments using one-way ANOVA with Tukey’s multiple comparison test (GraphPad Prism, version 9.1.1).

## Results

### Warming of immunoglobulins decreases viscosity

At 20 °C, the viscosity of immunoglobulins was shown to be dependent on concentration, with approximately 3 mPas for SCIG 10% and around 16 mPas for SCIG 20%. At 37 °C, viscosity is reduced. Importantly, this effect was more pronounced with SCIG 20% (~ 8 mPas, ~ 50% reduction) than with SCIG 10% (~ 2 mPas, ~ 33% reduction; Online Resource 1 [Suppl. Fig. 1]).

### In-line warming of SCIG 20% does not impact product quality

After passage of the immunoglobulin solution through the infusion warmer system (set at 38 °C), no changes in visual appearance (Online Resource 1 [Suppl. Table 1]), turbidity (Online Resource 1 [Suppl. Fig. 2]), hydrodynamic diameter, and polydispersity index (PDI) by dynamic light scattering (DLS) were detectable (Online Resource 1 [Suppl. Fig. 3]). A slight increase of the main monomer peak and a decrease of the dimer peak were detectable (Online Resource 1 [Suppl. Fig. 4]). The molecular size distribution of polyclonal IgG preparations showed a dynamic equilibrium between monomers, dimers, and polymers. Especially, dimerization was partly reversible. No change of main- and dimer-peak sum was detectable (Online Resource 1 [Suppl. Fig. 4]). Warming seemed to result in a drop in dimer content, which in turn leads to an increased monomer fraction. Furthermore, a slight reduction of aggregates, fragments (Online Resource 1 [Suppl. Fig. 5]), subvisible particles (≥ 10 μm), and subvisible particles (≥ 25 μm) was detectable (Online Resource 1 [Suppl. Fig. 6]).

The particle counts after the infusion warmer passage were below the light obscuration (LO)-subvisible particle United States Pharmacopeia limit [35] as point of orientation. Micro-flow imaging has been described by Sharma et al. [36] and Huang et al. [37] to be about one order of magnitude more sensitive for protein particles than LO. Overall, none of the executed tests (visual inspection, turbidity, DLS, SEC-HPLC, and MFI) showed relevant changes of the product due to the use of the infusion warmer device, beside the slight shift of the equilibrium between IgG-monomers and IgG-dimers. For all parameters, no impact of flow rate was found.

In summary, no relevant differences in physical quality parameters of SCIG 20% before and after passing the infusion warmer were observed. Thus, the product quality of SCIG 20% was not impacted using the infusion warmer system BW 685 S with connected TubeFlow (AF365F) at the investigated temperature and flow rates. In addition, the data indicate that back-pressure was not observed for the warming device (BW 685 S, Biegler), allowing for testing up to 7.5 mL/min flow rates.

### Facilitation and warming reduce in-line pressure during subcutaneous infusion into pigs compared to conventional SCIG 20%

#### Evaluation of a 19 G needle to reduce the impact of the needle resistance on in-line pressure

**Fig. 1:**
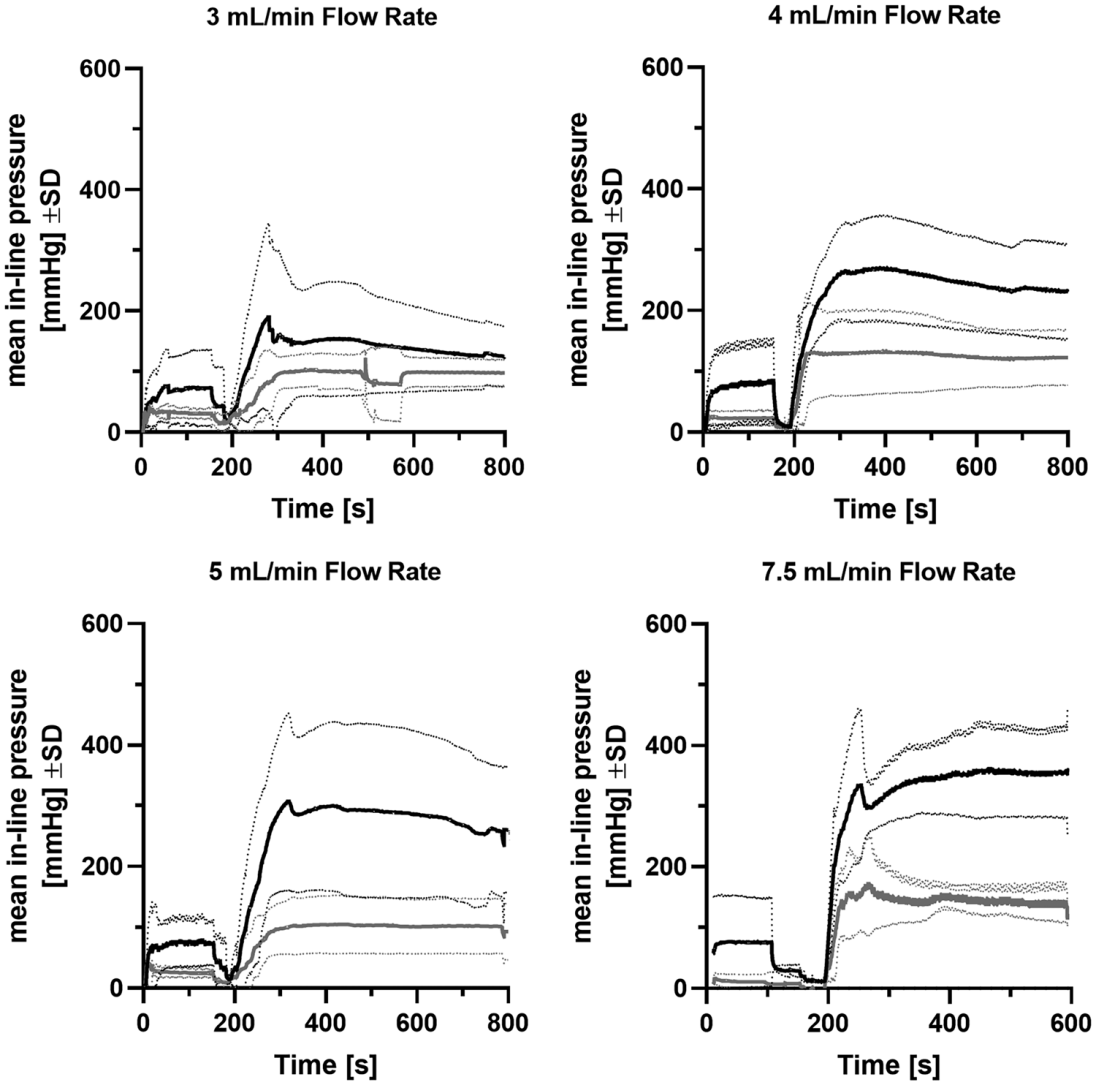
Comparison of mean in-line pressures for different SCIG preparations infused subcutaneously at different flow rates in pigs. Buffer (for conventional) or rHuPH20 (for facilitated) was infused from time point 0–200 s, followed by infusion of SCIG 20% at flowrates of 3, 4, 5, and 7.5 ml/min, starting at time point 200 s. Mean (full lines) in-line pressure ± standard deviation (dotted lines) is lower with warmed fSCIG 20% (gray) than with conventional SCIG 20% (black). The difference in mean maximum and mean in-line pressure was statistically significant for a flow rate of 5 mL/min and 7.5 mL/min (*p* > 0.033). f, facilitated; SCIG, subcutaneous immunoglobulin; rHuPH20, recombinant human hyaluronidase PH20

The SC administration of 50 mL of SCIG 20% at flow rates of 3, 4, 5, and 7.5 mL/min showed a distinct reduction in the in-line pressure with warmed fSCIG 20% compared to conventional, un-warmed, non-facilitated SCIG 20% (Fig. 1). A flow rate dependent increase in mean and maximum in-line pressures was observed with conventional SCIG 20%, whereas with warmed fSCIG 20%, in-line pressure remained essentially stable through up to the highest flow rate tested. At a flow rate of 3 mL/min, mean ± SD in-line pressure was 101.9 ± 25.8 mmHg with SCIG 20% vs. 92.8 ± 15.9 mmHg with warmed fSCIG 20%. Respective results were 234.6 ± 42.7 mmHg vs. 122.6 ± 31.8 mmHg at 4 mL/min, 298.9 ± 58.5 mmHg vs. 127.3 ± 10.2 mmHg at 5 mL/min, and 326.8 ± 38.51 mmHg vs. 139.8 ± 19.44 mmHg at 7.5 mL/min. The difference in mean maximum in-line pressure was statistically significant at flow rates of 5 mL/min (*p* = 0.045) and 7.5 mL/min (*p* = 0.0123). Fluid temperatures recorded at baseline (i.e., during infusion of buffer or rHuPH20) ranged from 16.16 to 25.10 °C. Using the in-line warming device, temperatures almost instantly increased to a plateau with a maximum of 29.59–35.95 °C, with the plateau being maintained for the entire infusion time. All low-range temperature measurements were recorded in the 7.5 mL/min cohort. This may be due to potentially different experimental conditions, as the cohort was treated on a separate day, and which is supported by the low baseline temperatures recorded (16.16–18.80 °C vs. 22.08–25.10 °C in other cohorts). As the difference between baseline and maximum temperature is comparable between cohorts, we do not consider the high flow rate and resulting shorter flow time in the warming device as a root cause for the lower plateau temperatures observed at 7.5 mL/min (29.59–30.54 °C vs. 31.66–35.95 °C).

Regarding comparison of in-line pressures during SC infusion at a flow rate of 5 mL/min of fSCIG 20% warmed to maximum temperatures from 34.25 to 34.61 °C and non-warmed fSCIG 10%, the mean ± SEM in-line pressure was 100.7 ± 23.1 mmHg with fSCIG 20% and 45.7 ± 10.0 mmHg with fSCIG 10%. The observed differences between the in-line pressures are in line with the differences in the viscosities of SCIG 10% (3 mPas) and in-line warmed SCIG 20% (8 mPas).

Local tolerance was assessed for all infusion sites of the treatment conditions described above. The blebs that initially formed during infusion decreased in size over time and were completely resolved after 24 h. The scores associated to the local reaction were comparable between the treatment approaches, with the highest values (score of 5–8) at the initial time-point and a progressive reduction to no signal (score of 0) after 24 h (Online Resource 1 [Suppl. Table 2, Suppl. Fig. 7]). The histopathological evaluation of infusion sites revealed acute inflammation in the SC tissue, muscle, and hypodermis, with an edema of mild to moderate severity. There were no differences in incidence and severity of local reactions in the experimental groups (Online Resource 1 [Suppl. Fig. 8]).

#### Evaluations of clinically relevant 23 G and 24 G needles

**Fig. 2:**
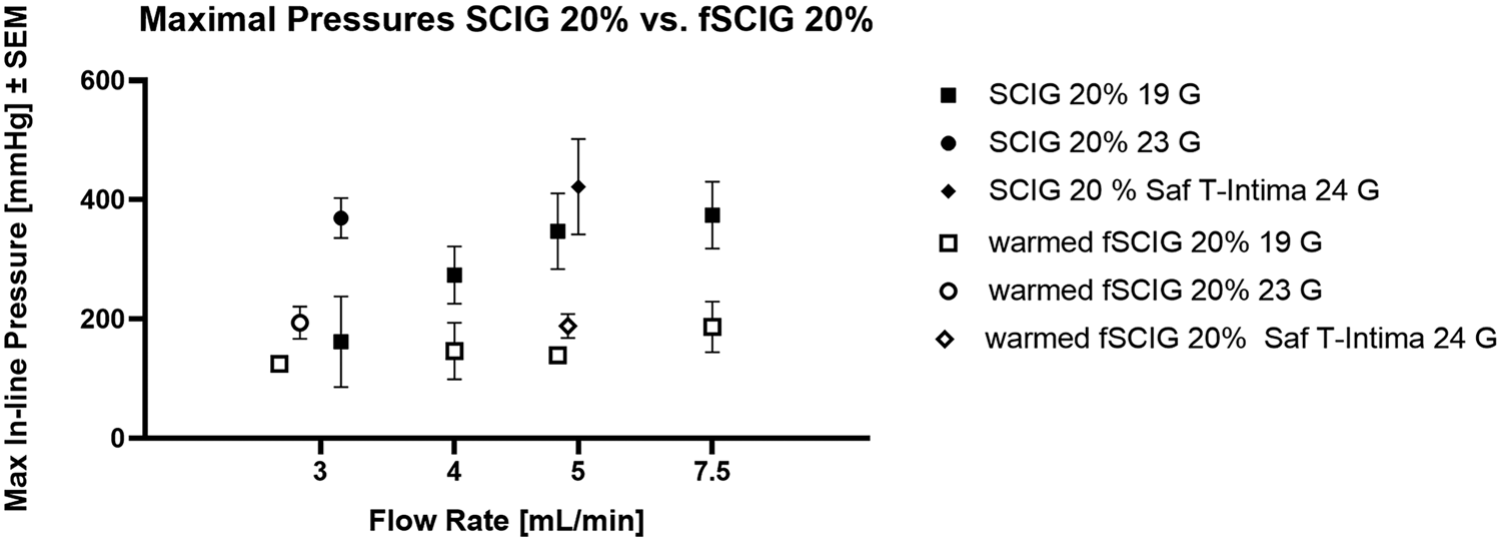
Summary of maximum in-line pressures with different treatment conditions. While warming and facilitation by rHuPH20 already showed a positive impact on maximum in-line pressures using 19 G needles which are commonly used in animal studies in pigs, this effect was much more pronounced using clinically relevant, smaller needle sizes of 23 G and 24 G. f, facilitated; G, gauge; SCIG, subcutaneous immunoglobulin (subcutaneous); rHuPH20, recombinant human hyaluronidase PH20

Additional SC infusion experiments were performed using clinically relevant needle sizes of 23 G and 24 G. Results showed clear differences between warmed fSCIG 20% versus non-warmed, conventional SCIG 20%. When a 23 G needle set was used at a flow rate of 3 mL/min, mean maximum in-line pressures were 193.6 mmHg vs. 369.0 mmHg (for warmed fSCIG 20% and conventional SCIG 20%, respectively; Fig. 2) and mean in-line pressures were 177.8 mmHg vs. 304.4 mmHg, while there were no differences between these treatments using a 19 G needle set (Fig. 2).

The BD-Saf T-intima 24 G infusion set was tested as a potential clinical infusion system. When using this system at a flow rate of 5 mL/min, mean maximum in-line pressures ± SEM were consistently and statistically significantly lower for fSCIG 20% (350.4 ± 32.2 mmHg) than for SCIG 20% (505.1 ± 37.4 mmHg; *p* = 0.035). Furthermore, warmed fSCIG 20% was infused using the BD-Saf T-intima 24 G set, showing consistently and statistically significantly lower mean maximum in-line pressures ± SEM for warmed fSCIG 20% (188.0 ± 20.0 mmHg) than for SCIG 20% (421.8 ± 80.2 mmHg; *p* = 0.0475; Fig. 2). Comparison of in-line pressures during SC infusion of fSCIG 20% (3 mL/min) with either the BD-Saf T-intima 24 G needle set or a Venofix 19 G needle showed no statistically significant differences (*p* = 0.3400). Mean maximum in-line pressures ± SEM were 163.7 ± 28.4 mmHg for BD-Saf T-intima 24 G and 125.1 ± 21.6 mmHg for Venofix 19 G, respectively (Fig. 2).

#### Ramp up study that simulates potential clinical dosing regimen

**Fig. 3:**
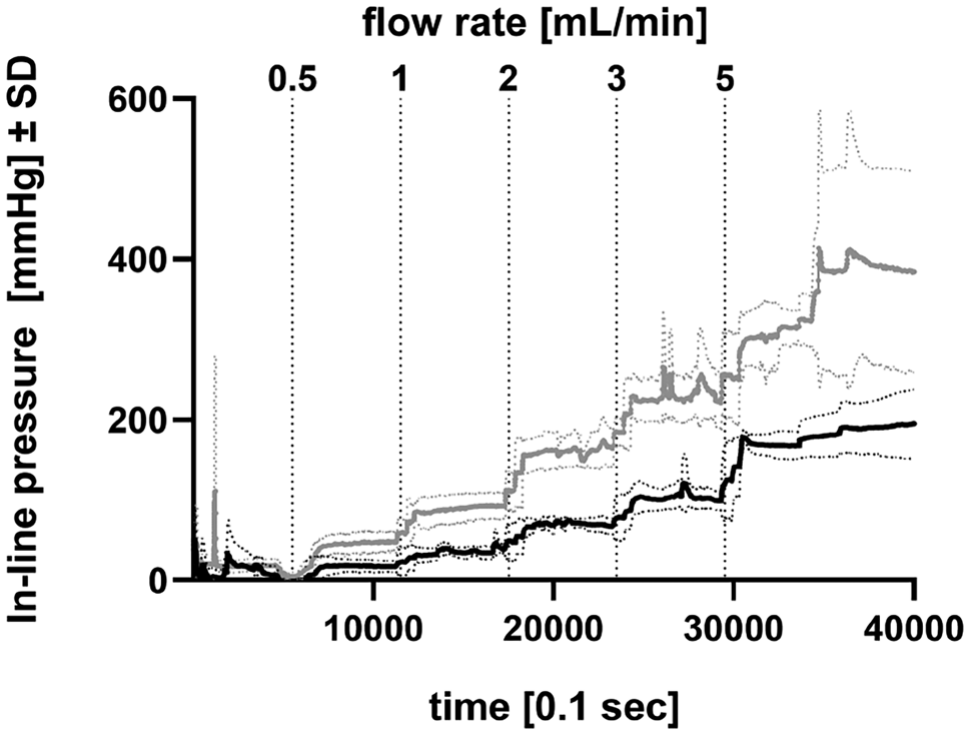
Comparison of mean in-line pressures of different SCIG preparations during subcutaneous infusion after ramp up of flow rates in pigs. rHuPH20 was infused from time point 0–480 s, followed by infusion of SCIG, starting at time point 480 s at 0.5 mL/min. The flow rate was increased every 600 s (0.5 mL/min, 1 mL/min, 2 mL/min, 3 mL/min, 5 mL/min). For all flow rates, mean (full lines) in-line pressure ± standard deviation (dotted lines) is lower with warmed fSCIG 20% (black) than with non-warmed fSCIG 20% (gray). The difference in mean maximum and mean in-line pressure was statistically significant for a flow rate of 2 mL/min (*p* = 0.0077) and 3 mL/min (*p* = 0.0153). f, facilitated; SCIG, subcutaneous immunoglobulin (subcutaneous); rHuPH20, recombinant human hyaluronidase PH20

To mirror a potential clinical dosing regimen, an in vivo ramp up study with increasing flow rates of 0.5–5 mL/min was performed. In-line pressure increased with increasing flow rates for both in-line warmed fSCIG 20% and non-warmed fSCIG 20%. The increase was much less pronounced with warmed fSCIG 20% (Fig. 3).

At a flow rate of 0.5 mL/min, mean maximum ± SEM in-line pressure was 24.7 ± 9.1 mmHg with warmed fSCIG 20% vs. 58.6 ± 16.9 mmHg with fSCIG 20%. Respective results were 57.7 ± 12.5 mmHg vs. 113.4 ± 24.0 mmHg at 1 mL/min, 86.8 ± 11.1 mmHg vs. 190.3 ± 9.9 mmHg at 2 mL/min, 141.8.0 ± 26.0 mmHg vs. 320.8.0 ± 16.4 mmHg at 3 mL/min, and 206.9 ± 21.4 mmHg vs. 482.6 ± 127.1 mmHg at 5 mL/min. The difference in mean maximum in-line pressure was statistically significant at flow rates of 2 mL/min (*p* = 0.0077) and 3 mL/min (*p* = 0.0153).

Regarding local tolerance, the blebs that initially formed during infusion decreased in size over time and were completely resolved after 24 h. The scores associated to the local reaction were comparable between the treatment approaches, with the highest values (score of 6–8) at the initial time-point and a progressive reduction to low signals (score of 0–1) after 24 h (Online Resource 1 [Suppl. Table 3, Suppl. Figs. 8 and 9]). The histopathological evaluation of infusion sites revealed acute inflammation consisting mainly of neutrophil polymorphonuclear leukocytes and lymphocytes in the SC tissue and hypodermis, with an edema of mild to moderate severity.

### Warming of SCIG 20% does not influence PK parameters

**Fig. 4:**
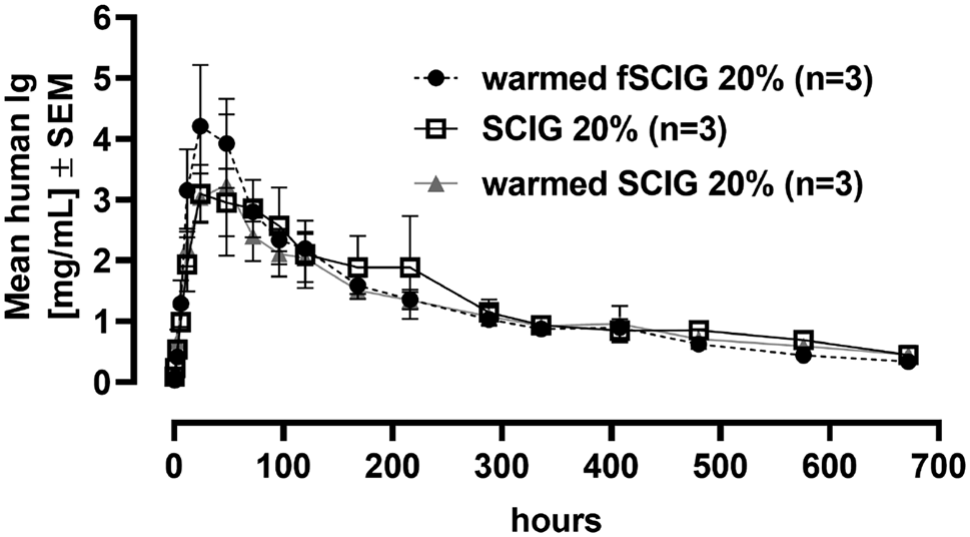
Pharmacokinetics of fSCIG 20% and warmed SCIG 20% versus conventional SCIG 20%. A trend for increased C_max_, indicative of increased dispersion and absorption, was observed with warmed fSCIG 20%. All other parameters were not influenced by warming and facilitation. Warming alone did not impact the pharmacokinetic profile of SCIG 20%. C_max_, maximum concentration; f, facilitated; SCIG, immunoglobulin (subcutaneous)

The PK parameters after SC infusion of warmed fSCIG 20%, non-warmed conventional SCIG 20%, and warmed conventional SCIG 20% are summarized in Table 2 and Fig. 4. The pharmacokinetic profiles of in-line warmed and non-warmed SCIG 20% infused at flow rates of 5 and 1 mL/min, respectively, were similar. In comparison to the in-line warmed treatment approaches, the prolonged non-warmed SCIG 20% infusion period of about 45 min was not reflected in a higher time to C_max_ value (T_max_) due to the high inter-individual variability and the selected blood sampling timepoints at relevant time frame. Pharmacokinetic parameters of in-line warmed fSCIG 20% infused at a flow rate of 5 mL/min were comparable with conventional SCIG 20%, with a trend towards higher maximum plasma concentration (C_max_), T_max_, and exposure as assessed by area under the concentration–time curve from time 0 until the last quantifiable concentration. Statistical analysis did not reveal significant differences between the three approaches for C_max_, T_max_, and AUC_last_.

**Table 2:** Pharmacokinetic parameters of human immunoglobulins in serum after subcutaneous infusion in pigs

**Parameter**	**fSCIG 20% (warmed)**	**SCIG 20% (non-warmed)**	**SCIG 20% (warmed)**
Flow rate (mL/min)	5	1	5
*N*	3	3	3
C_max_ (mg/mL)	4.9 ± 1.5	3.8 ± 0.7	3.6 ± 1.5
T_max_ (hr)	48 ± 24.0	40 ± 13.9	40 ± 13.9
t_1/2_ (hr)	183.7 ± 19.1	192.3 ± 5.6	218.4 ± 57.2
AUC_0-last_ (mg·hr/mL)	813.8 ± 109.1	775.1 ± 266.5	759.7 ± 239.0

*AUC*_*0-last*_ area under the concentration–time curve from time 0 until the last quantifiable concentration (672 h), *C*_*max*_ maximum concentration, *f* facilitated, *hr* hours, *SCIG 20%* subcutaneous immunoglobulin infusion 20% (human); *t*_*1/2*_, half-life; *T*_*max*_, time to reach maximum concentration; mean ± SD (median for T_max_)

## Discussion

Low-concentration (10%) immunoglobulin for SC infusion requires large volumes to deliver the required dose of immunoglobulins and thus requires long infusion times. High viscosity is a limiting factor in the use of high-concentration (20%) immunoglobulin for SC infusion, as high viscosity limits infusion flow rates due to flow resistance deriving from the administration system, mainly from the needle, which lengthens infusion time. Use of rHuPH20 allows administration of larger volumes of SCIG, has a potential to improve bioavailability of SCIG 20% compared to conventional SCIG, allowing the same dosing as for IVIG, and thus enabling less frequent infusions. However, rHuPH20 has no impact on product viscosity.

In liquids, dynamic viscosity is inversely proportional to temperature. In the presented studies, we tested the hypothesis that warming a fSCIG 20% solution could circumvent the limitation of higher viscosity.

In vitro assessment confirmed a lower viscosity of SCIG 20% at higher temperatures. An in vitro feasibility study using in-line warming of SCIG 20% was conducted to evaluate potential impacts on product quality as assessed by micro-flow imaging, dynamic light scattering, visual appearance, turbidity, and SEC-HPLC. In-depth characterization of in-line warmed SCIG 20% showed that there were no negative effects of warming on product quality. A slight shift of the equilibrium between IgG-monomers and IgG-dimers was observed in in-line warmed SCIG 20%, which could even be considered a positive effect for product quality. During accelerated stability testing for CUVITRU, SCIG 20% was exposed to a temperature of 40 °C. No impact on quality parameters assessing denaturation, aggregation, and fragmentation as well as antibody function were detected after 1 month, which is significantly longer than the exposure to elevated temperatures during infusion with the warming device. The setup was then tested in SC infusion studies in pigs. Based on previous experience, the pig model is considered the most suitable model to assess feasibility of SC administration of immunoglobulins [33]. Results of in vivo studies showed that SC infusion of in-line warmed fSCIG 20% was feasible at flow rates of 3, 4, 5, and 7.5 mL/min, with a distinct reduction of maximum and mean in-line pressure compared to conventional SCIG 20% (Fig. 1), and that local reactions were comparable to fSCIG 10%. The pharmacokinetic profiles of in-line warmed and non-warmed SCIG 20% infused at flow rates of 5 and 1 mL/min, respectively, were similar. Pharmacokinetic parameters of in-line warmed fSCIG 20% infused at a flow rate of 5 mL/min were comparable with conventional SCIG 20%, with a trend towards higher C_max_, T_max_, and AUC_0-tlast_ (Table 2, Fig. 4) indicative of increased dispersion. These initial experiments further supported the hypothesis tested.

Additional in vivo experiments were conducted using clinically relevant, smaller needle sizes of 23 G and 24 G, compared to 19 G used in the first set of experiments. Interestingly, the effects of facilitation by rHuPH20 as well as of warming of fSCIG 20%, were much more pronounced with smaller needle sets (Fig. 3), leading to significantly reduced in-line pressures compared with non-warmed conventional SCIG 20%. This is considered to be caused by the increase of drag as the difference in diameters of the line and the needle set increases. Drag not only depends on geometry of the tube, but also depends on viscosity of the fluid. Thus, using a smaller needle diameter may have increased the significance of lower viscosity. Based on these observations, it may be hypothesized that the beneficial effect of in-line warming of fSCIG 20% could be greater in a clinical setting compared to a standard animal model setting. As it is generally assumed that needles of smaller diameter cause less sensation of pain at needle insertion, feasibility of SC infusion of fSCIG 20% with 23 or 24 G needle sets was an important endpoint in the pre-clinical studies presented.

In a study in healthy individuals, perceived pain immediately after needle insertion (*n* = 144) and at the end of a 2 or 3 mL SC injection did not markedly differ, nor were there any significant differences between the two injection volumes tested (*p* = 0.89), indicating that needle insertion itself is perceived more uncomfortably than the following SC injection [38], supporting the theory that frequent infusions are to be avoided to increase patient compliance.

Injection rate does not seem to impact injection-related pain in SC administration [39]. In the study by Berteau et al. [38], there were no significant differences in perceived pain between injections at 0.02 mL/s and 0.30 mL/s (*p* = 0.79). Dias et al. [40], despite reporting statistically significant increase in perceived pain with increased injection rate (1.2 mL bolus, *n* = 48; 3.5 mL/min, *n* = 48; 3.5 mL/4 min, *n* = 48; 3.5 mL/10 min, *n* = 46) in a randomized, crossover study in healthy subjects, considered this result as irrelevant, as the differences in values (6.8 mm, 5.6 mm) were below a clinically meaningful difference on a 100 mm visual analog score (i.e., 13 mm). These findings reported in the literature support the theory that higher flow rates facilitating shorter infusion times might improve patient experience.

Interestingly, two independent studies report higher viscosity of SC administered fluids to be associated with less pain compared with low-viscosity fluids [38, 41]. Viscosity of administered fluids was categorized into 1 mPas, 8–10 mPas, and 15–20 mPas, with fluids of 15–20 mPas being most easily tolerated (*p* = 0.0003 vs. 1 mPas) [38]. Warmed fSCIG 20% (at 37 °C) was shown to have an intermediate dynamic viscosity of around 8 mPas. Infusion of both warmed and non-warmed fSCIG 20% is thus expected to be well tolerated in terms of perceived pain.

Our studies show advantages in ease of infusion of in-line warmed fSCIG 20% over fSCIG 20%, as indicated by lower in-line pressures.

The lower in-line pressures would thus likely increase the performance of the administration system in daily practice, including less pump alarms, and the use of smaller diameter needles in combination with weaker pumps; on the other hand, the setup of the in-line warming device may be somewhat challenging, especially in a home-treatment setting.

Our studies showed that SC infusion was well tolerated locally also without warming, up to an infusion flow rate of 5 mL/min as local site reactions were comparable between in-line warmed fSCIG 20%, non-warmed fSCIG 20%, and fSCIG 10%. Future studies will have to address the technical advantages and disadvantages of in-line warming versus non-warmed fSCIG 20% during infusion, as assessed by ease of handling and pump performance, as well as to further assess the impact on tolerability. Importantly, clinical and human factor evaluations will be required to assess the balance of the beneficial effects of a warming to device in relation to the burden of an additional device needed for administration.

As a next step, various infusion rates of fSCIG 20% (TAK-881) with and without in-line warming have recently been tested in a phase 1, single-dose, open-label, three-arm study in healthy adult subjects (NCT05059977). The primary endpoint was tolerability; secondary outcome measures included infusion time and safety. Results were pending at submission of this manuscript. If successful, SC infusion of fSCIG 20% allowing for reduced volumes and shorter infusion could contribute to a more patient-centric routine treatment of primary and secondary immunodeficiency, as well as autoimmune diseases.

## Supplementary Information

Below is the link to the electronic supplementary material.

Supplementary file1 (PDF 2.75 MB)

## Data Availability

The datasets generated during and/or analyzed during the current study are available from the corresponding author on reasonable request.
