# Supplementary material for: In-line warming reduces in-line pressure of subcutaneous infusion of concentrated immunoglobulins
Source: Drug Deliv Transl Res. 2023 Mar 15;13(9):2367–77. doi: 10.1007/s13346-023-01321-y (PMC10382333; doi:10.1007/s13346-023-01321-y)
Supplement: Supplementary file 1 — Supplementary file1 (PDF 2.75 MB) [file 13346_2023_1321_MOESM1_ESM.pdf]

Supplementary Materials:
In-Line Warming reduces in-line Pressure of Subcutaneous Infusion of Concentrated Immunoglobulin

Journal: Drug Delivery and Translational Research

Peter Leidenmühler[1], Joris Höffinghoff[2], Norbert Haider[2], Gerald Brachtl[2], Markus Weiller[1], Ivan Bilic[1], Bagirath Gangadharan[1]

[1]Baxalta Innovations GmbH, a Takeda company, Vienna, Austria

[2]Baxter AG, a Takeda company, Vienna, Austria

Correspondence and requests for reprints should be addressed to Peter Leidenmühler, Donau City Strasse 7, A-1220 Vienna or peter.leidenmuehler@takeda.com.

Suppl. Figure 1: Dynamic Viscosity of Immunoglobulins at Different Temperatures

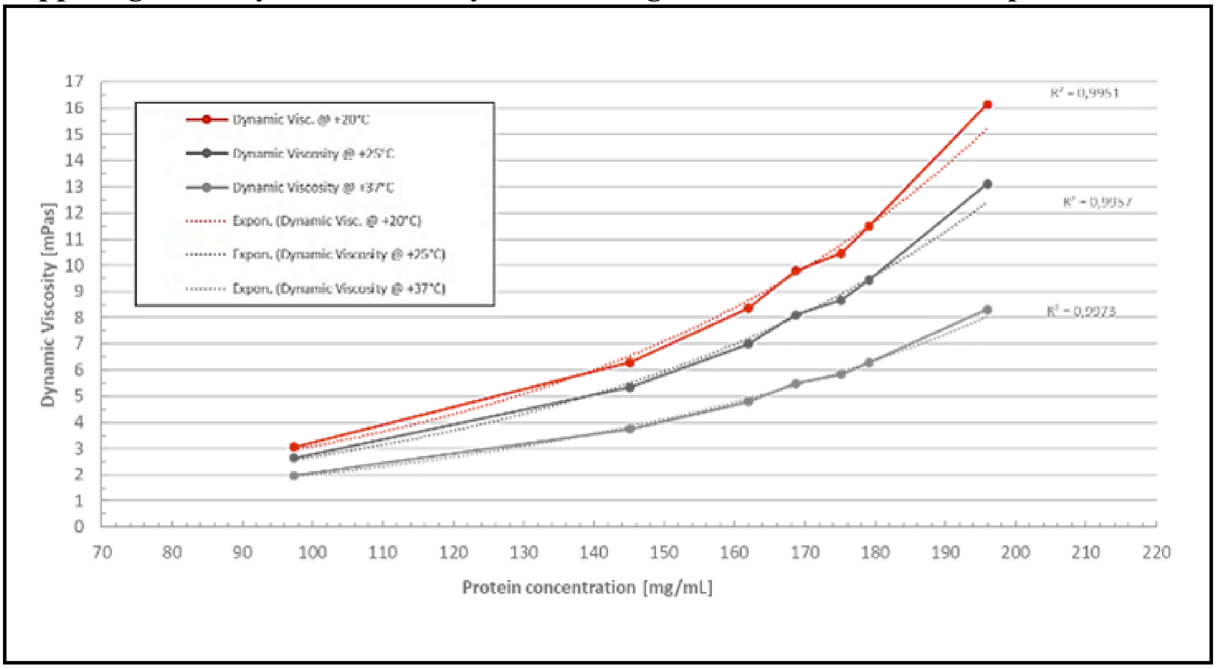

Dynamic viscosity (y-axis) of immunoglobulin preparations with increasing protein concentration (x-axis) at different temperatures (red = +20°C; dark grey = +25°C; light grey = +37°C). Dotted lines represent computed exponential increase.

Suppl. Table 1: Visual Appearance

Flow Rate (mL/min)	Infusion Warmer	Particles	Turbidity
2.5	before	A	2
	after	A	2
3.5	before	A	2
	after	A	2
4.5	before	A	2
	after	A	2
5.5	before	A	2
	after	A	2
6.5	before	A	2
	after	A	2
7.5	before	A	2
	after	A	2

Scoring: Particles (A = no particles visible; B = small single particles, barely visible; C = small single particles, easily visible; D = many particles, easily visible; E = particles ≥ 1 mm visible). Clearness (1 = clear solution; 2 = very slight turbidity; 3 = slight turbidity; 4 = cloudy solution; 5 = very turbid solution).

Suppl. Figure 2: Turbidity at UV 350 nm

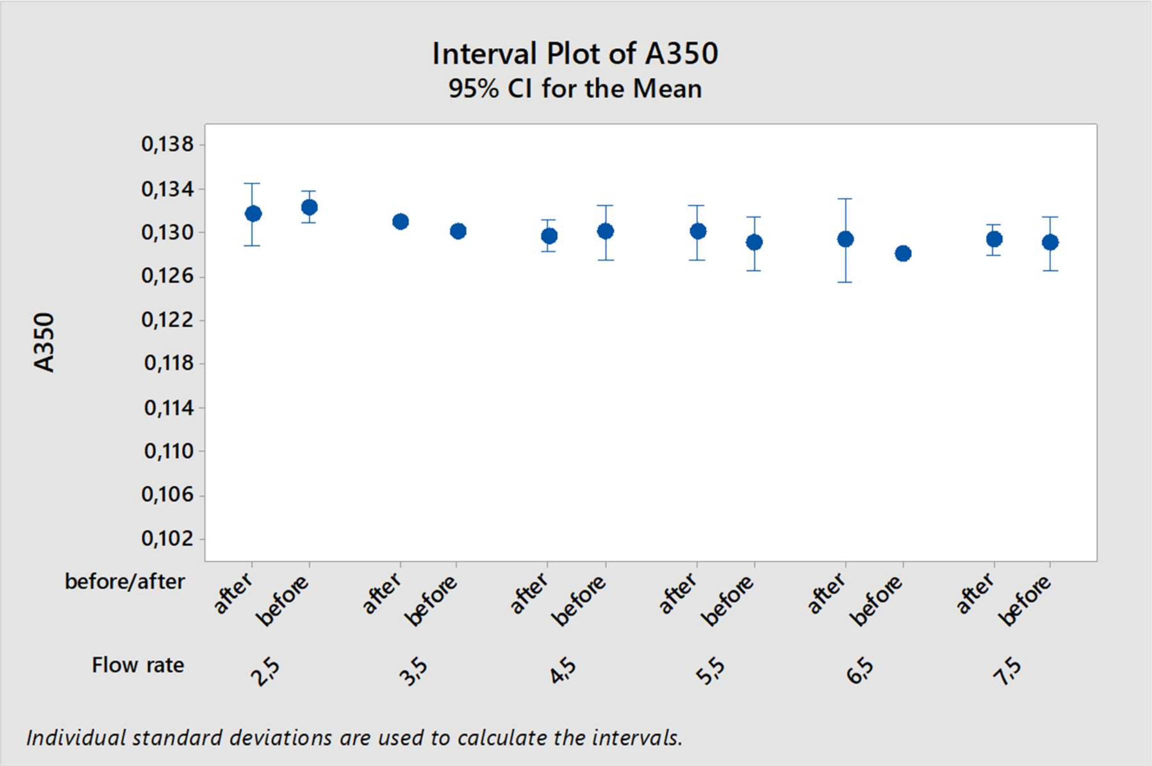

Turbidity at UV 350 nm of IgG solution before and after contact with infusion warmer at different flow rates. No marked changes after in-line warming were observed.

Suppl. Figure 3: Dynamic Light Scattering & Hydrodynamic Diameter and Polydispersity Index

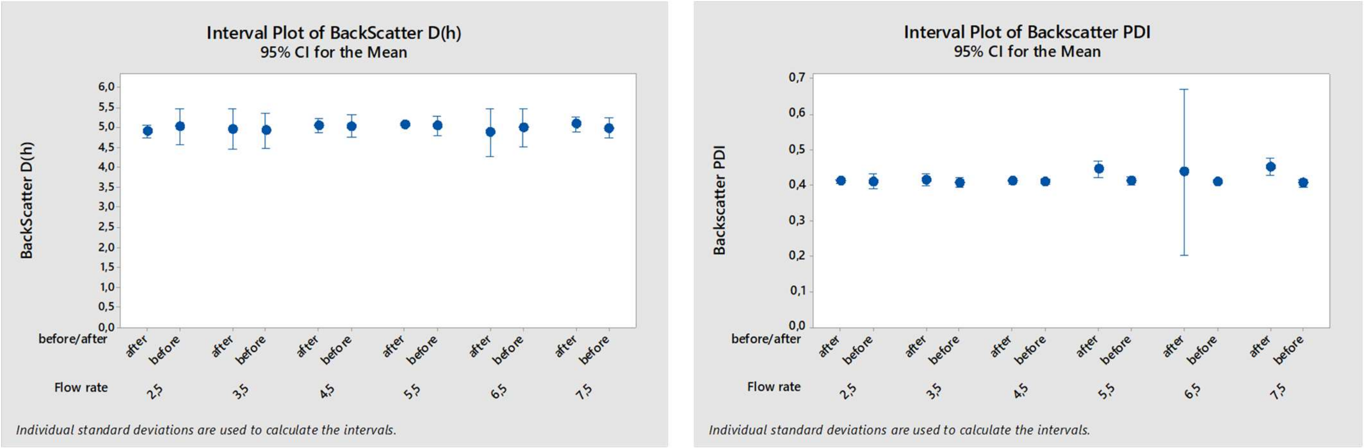

Dynamic light scattering (**left panel**) and polydispersity index (**right panel**) of IgG solution before and after contact with infusion warmer at different flow rates. No marked changes after in-line warming were observed.

Suppl. Figure 4: Size Exclusion Chromatography: Main- and Dimer-Peak

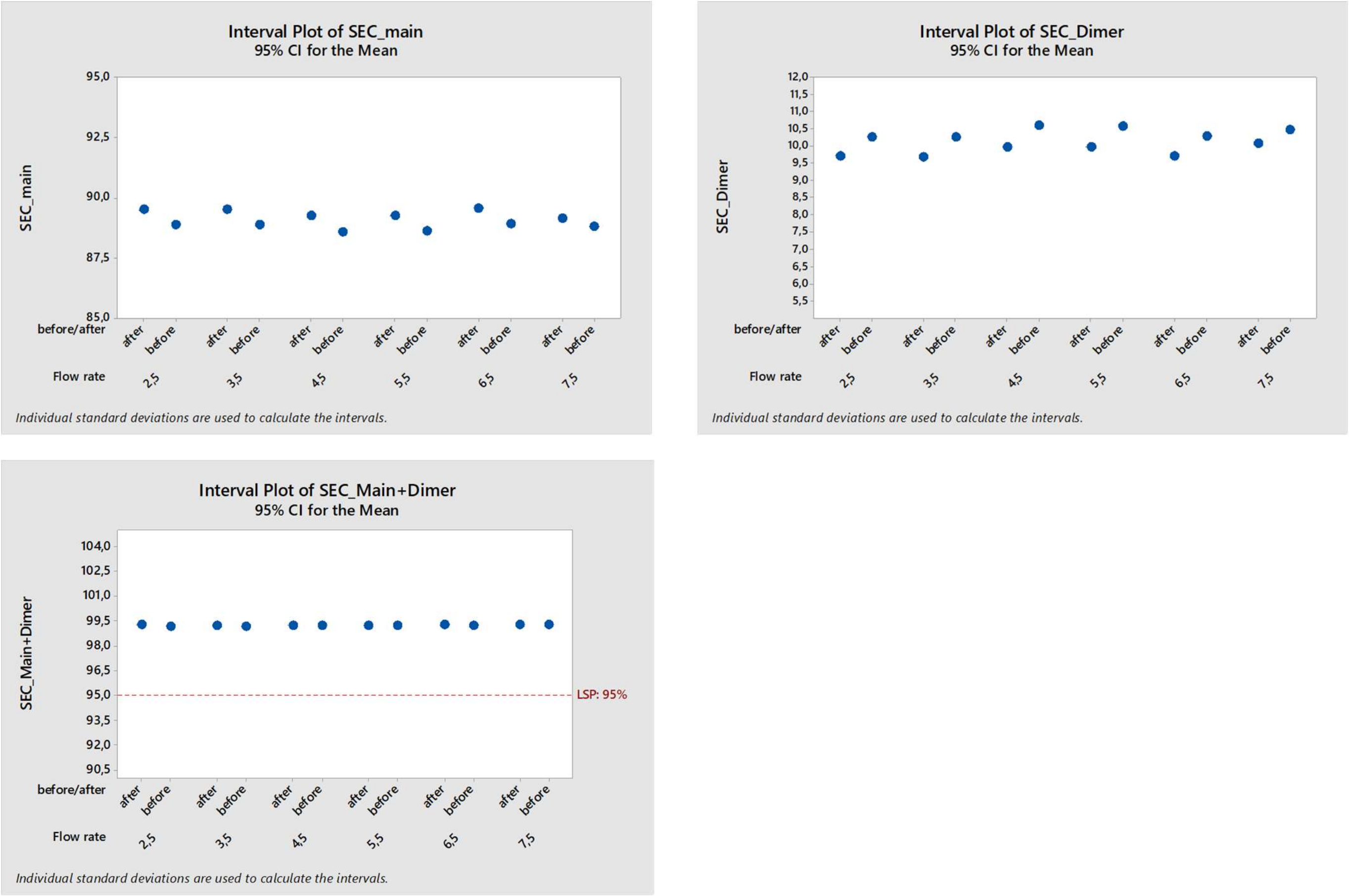

Top left panel: Size exclusion chromatography (SEC)-main peak of IgG solution before and after contact with infusion warmer at different flow rates. An increase in monomer content after in-line warming was observed. **Top right panel:** SEC-main (monomer) peak of IgG solution before and after contact with infusion warmer at different flow rates. A decrease in dimer content after in-line warming was observed. **Bottom left panel:** SEC-main and dimer peak before and after contact with infusion warmer at different flow rates. No changes were obsersved.

Suppl. Figure 5: Size Exclusion Chromatography: Aggregate- and Fragment-Peak

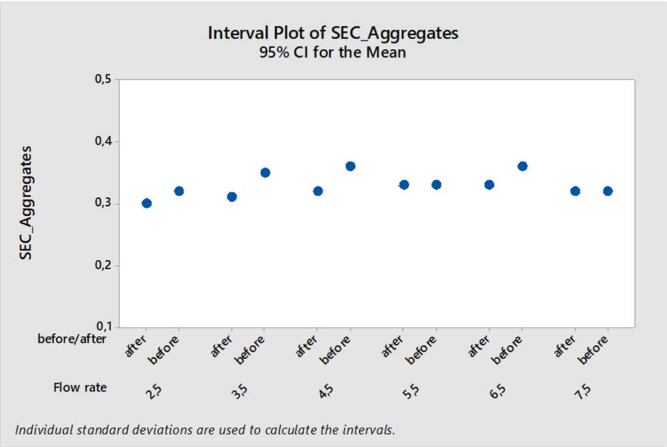

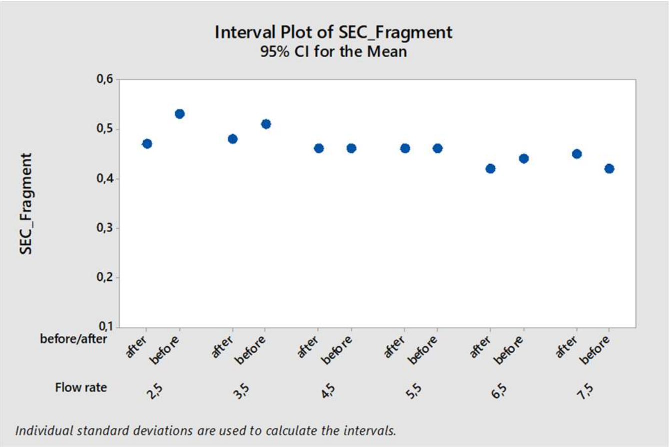

Left panel: Size exclusion chromatography (SEC)-main peak of IGG solution before and after contact with infusion warmer at different flow rates. A decrease or no changes in aggregate content after in-line warming were observed. **Right panel:** SEC-main peak of IGG solution before and after contact with infusion warmer at different flow rates. A decrease or no marked changes in fragment content after in-line warming were observed.

Suppl. Figure 6: Micro-flow Imaging

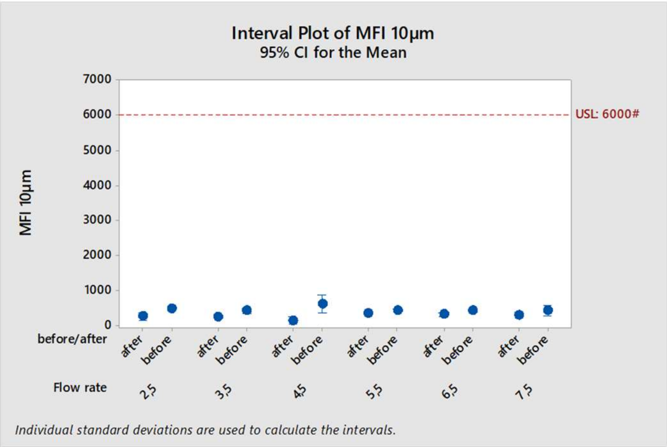

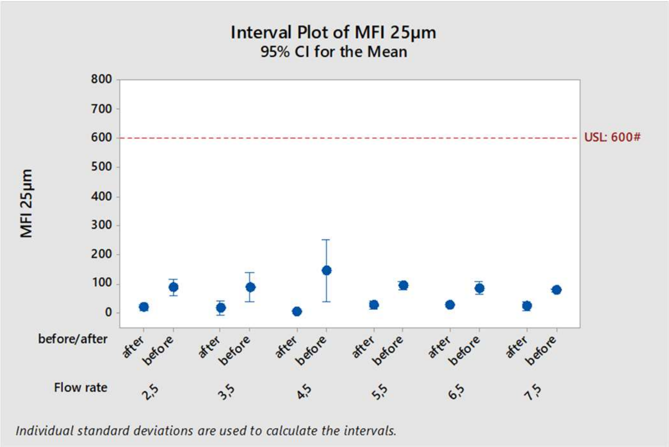

Micro-flow imaging of IGG solution before and after contact with infusion warmer at different flow rates showed a decrease or nor marked changes in subvisible particle content (**left panel:** 10 µm; **right panel:** 25 µm).

Suppl. Table 2: Size Measurements of Infusion-Site Blebs in Pigs after Subcutaneous Infusion of 50 mL fSCIG 10% or 20%

	fSCIG 10%, 50 mL at 5 mL/min				fSCIG 20%, 50 mL at 5 mL/min			
**Time Point (h)**	**0**	**2**	**4**	**24**	**0**	**2**	**4**	**24**
**Width (mm)**	59.49±9.91	54.17±13.34	46.49±7.07	0.0±0.0	62.38±1.70	60.81±5.76	58.03±0.88	0.0±0.0
**Length (mm)**	80.32±5.27	60.45±8.42	57.29±10.18	0.0±0.0	74.49±5.03	87.10±15.36	71.58±5.03	0.0±0.0
**Height (mm)**	29.09±2.26	12.34±1.24	12.12±6.90	0.0±0.0	30.21±11.30	18.15±6.26	17.06±6.51	0.0±0.0
**Surface (cm^2)**	37.68±8.71	26.16±9.91	21.20±6.90	0.0±0.0	36.46±1.47	41.95±11.27	32.61±1.80	0.0±0.0
**Volume (cm^3)**	73.72±22.56	21.92±10.31	17.59±8.38	0.0±0.0	72.87±24.50	48.29±3.86	36.68±12.11	0.0±0.0
**Local Reaction Score**	8±0	2±0	2±0	0±0	8±2.1	4±0	3±0.7	0±0

Means ± standard deviations are presented.

Suppl. Figure 7: Infusion-Site Blebs in Pigs after Subcutaneous Infusion 150 mL fIGSC 10% and warmed fIGSC 20%

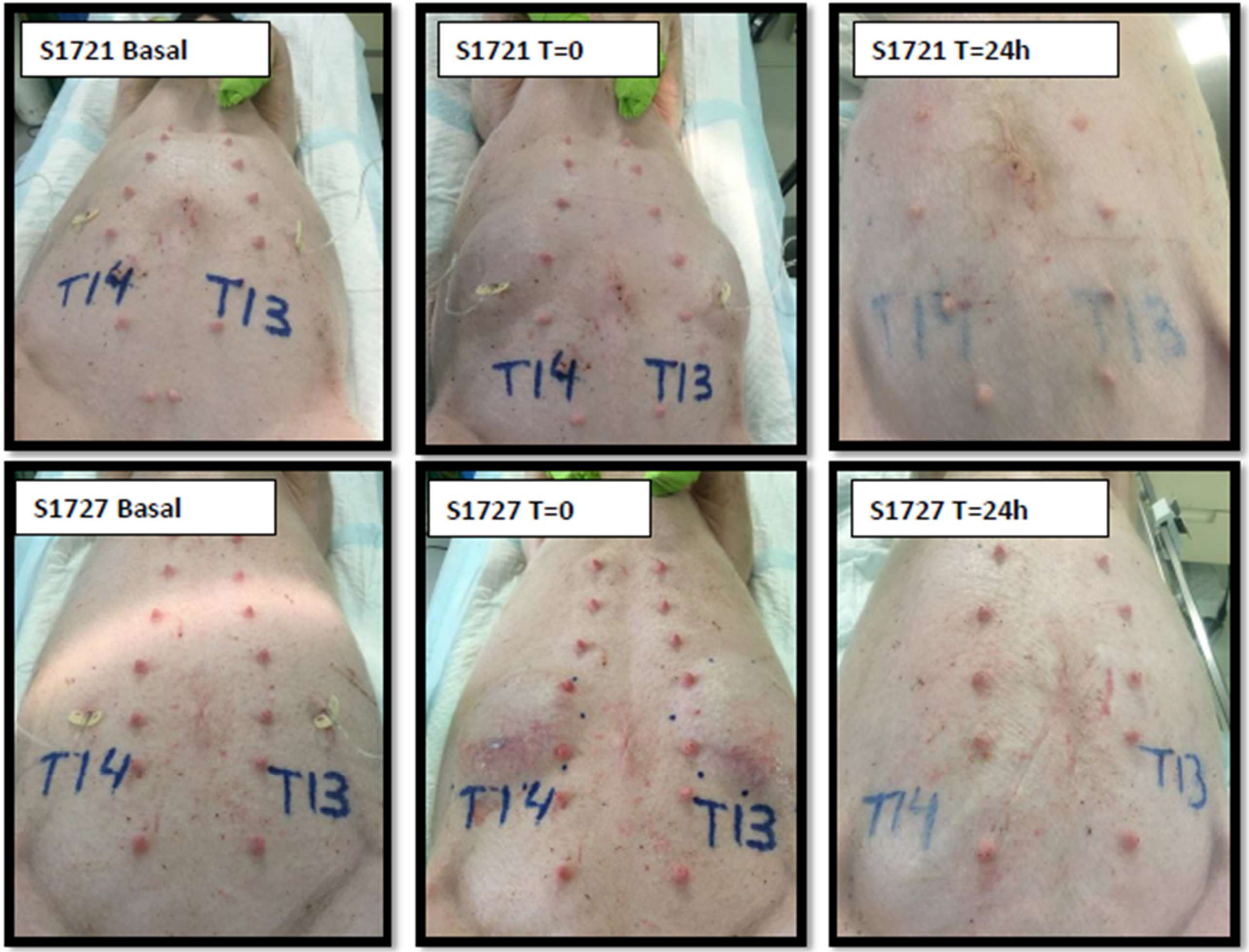

TI3 = 150 mL fIGSC 10%; TI4 = 150 mL in-line warmed fIGSC 20%

Suppl. Figure 8: Histopathology Assessment of Local Tolerability of SCIG Preparations in Pigs

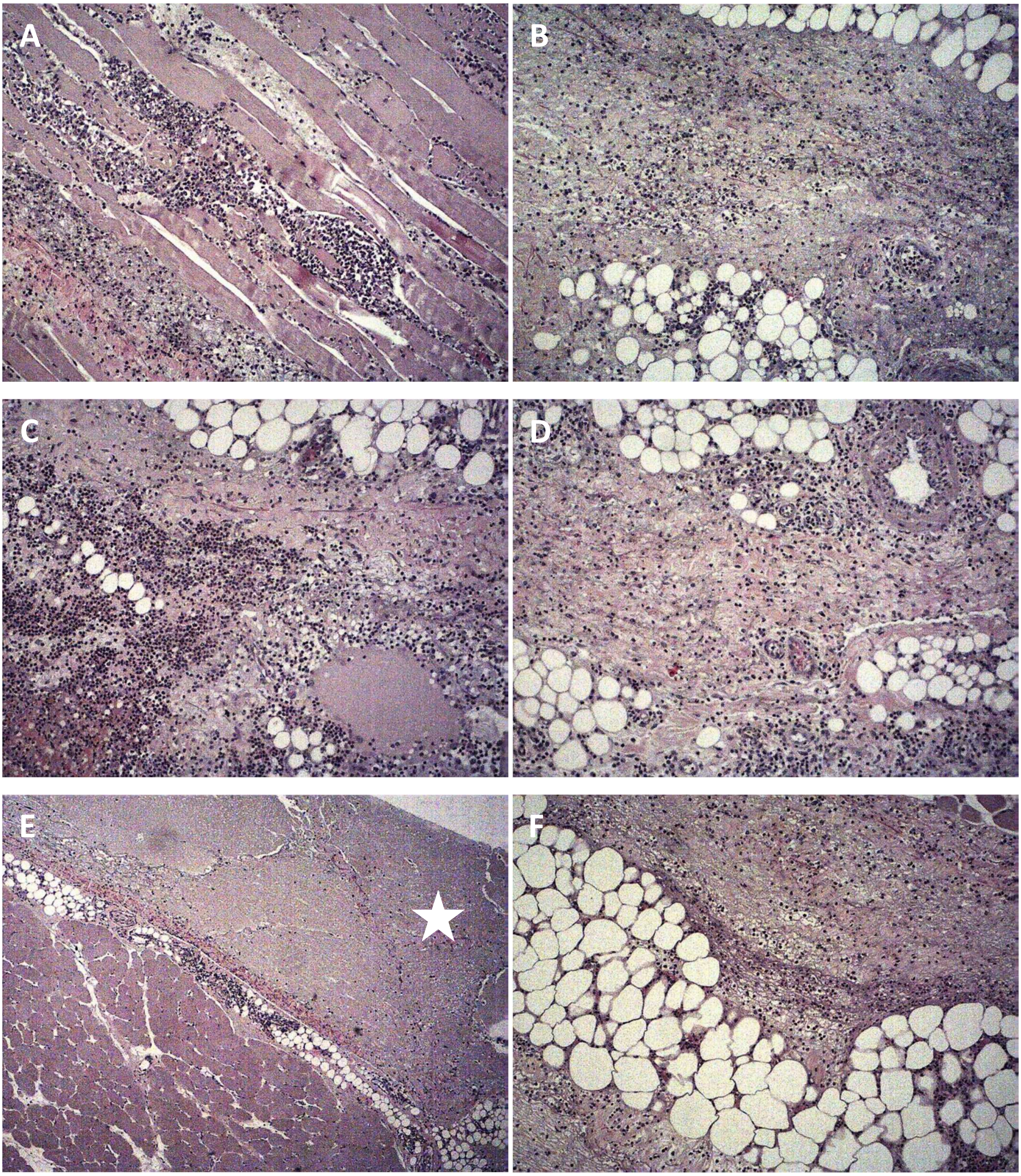

A. Buffer + 50 mL IGSC 20% (5 mL/min). Acute inflammation of muscular layer (Grade 3). H&E, Obj. 10x. **B.** 50 mL In-line warmed fIGSC 20% (5 mL/min). Acute inflammation of hypodermis (Grade 3). H&E, Obj. 10. **C.** 150 mL fIGSC 10% (5 mL/min). Acute inflammation of hypodermis (Grade 3). H&E, Obj. 10. **D.** 50 mL fIGSC 10% (5 mL/min). Acute inflammation of subcutaneous tissue (Grade 2). H&E, Obj. 10x. **E.** 150 mL in-line warmed fIGSC 20% (5 mL/min). Subcutaneous edema (Grade 3), marked with a star. H&E, Obj. 4x. **F.** 150 mL in-line warmed fIGSC 20% (5 mL/min). Acute inflammation of subcutaneous tissue (Grade 2). H&E, Obj. 10x.

Suppl. Table 3: Size Measurements of Infusion-Site Blebs in Pigs after Subcutaneous Infusion of 160 mL warmed and non-warmed fSCIG 20%

ID		fSCIG 20%, 160 mL at 5 mL/min							
	**Time Point (h)**	**0**		**2**		**4**		**24**	
	**In-Line Warming**	**Y**	**N**	**Y**	**N**	**Y**	**N**	**Y**	**N**
**S1890**	**Width (mm)**	106.42	110.56	98.96	107.33	83.51	113.52	0.00	0.00
	**Length (mm)**	125.95	132.38	108.85	118.48	107.16	98.99	0.00	0.00
	**Height (mm)**	30.00	27.58	22.93	20.93	23.57	11.13	0.00	0.00
	**Surface (cm²)**	105.27	114.95	84.60	99.87	70.28	88.26	0.00	0.00
	**Volume (cm³)**	210.54	211.36	129.33	139.36	110.44	65.49	0.00	0.00
	**Local Reaction Score**	7	7	5	5	3	1	1	1
**S1894**	**Width (mm)**	93.88	86.65	91.03	86.60	99.14	94.46	0.00	0.00
	**Length (mm)**	99.35	124.82	64.48	101.03	117.20	135.94	0.00	0.00
	**Height (mm)**	32.82	28.85	10.50	32.20	24.31	19.75	0.00	0.00
	**Surface (cm²)**	73.25	84.95	46.12	68.72	91.24	100.85	0.00	0.00
	**Volume (cm³)**	160.28	163.38	32.28	147.51	147.90	132.79	0.00	0.00
	**Local Reaction Score**	8	8	4	4	3	3	1	1
**S1895**	**Width (mm)**	94.43	-	82.50	-	83.70	-	0.00	-
	**Length (mm)**	129.57	-	99.52	-	97.95	-	0.00	-
	**Height (mm)**	33.46	-	43.98	-	28.17	-	0.00	-
	**Surface (cm²)**	216.36	-	64.48	-	64.39	-	0.00	-
	**Volume (cm³)**	96.10	-	189.07	-	120.92	-	0.00	-
	**Local Reaction Score**	6	-	4	-	2	-	1	-

Suppl. Figure 8: Infusion-Site Blebs in Pigs after Subcutaneous Infusion 160 mL warmed and non-warmed fSCIG 20%

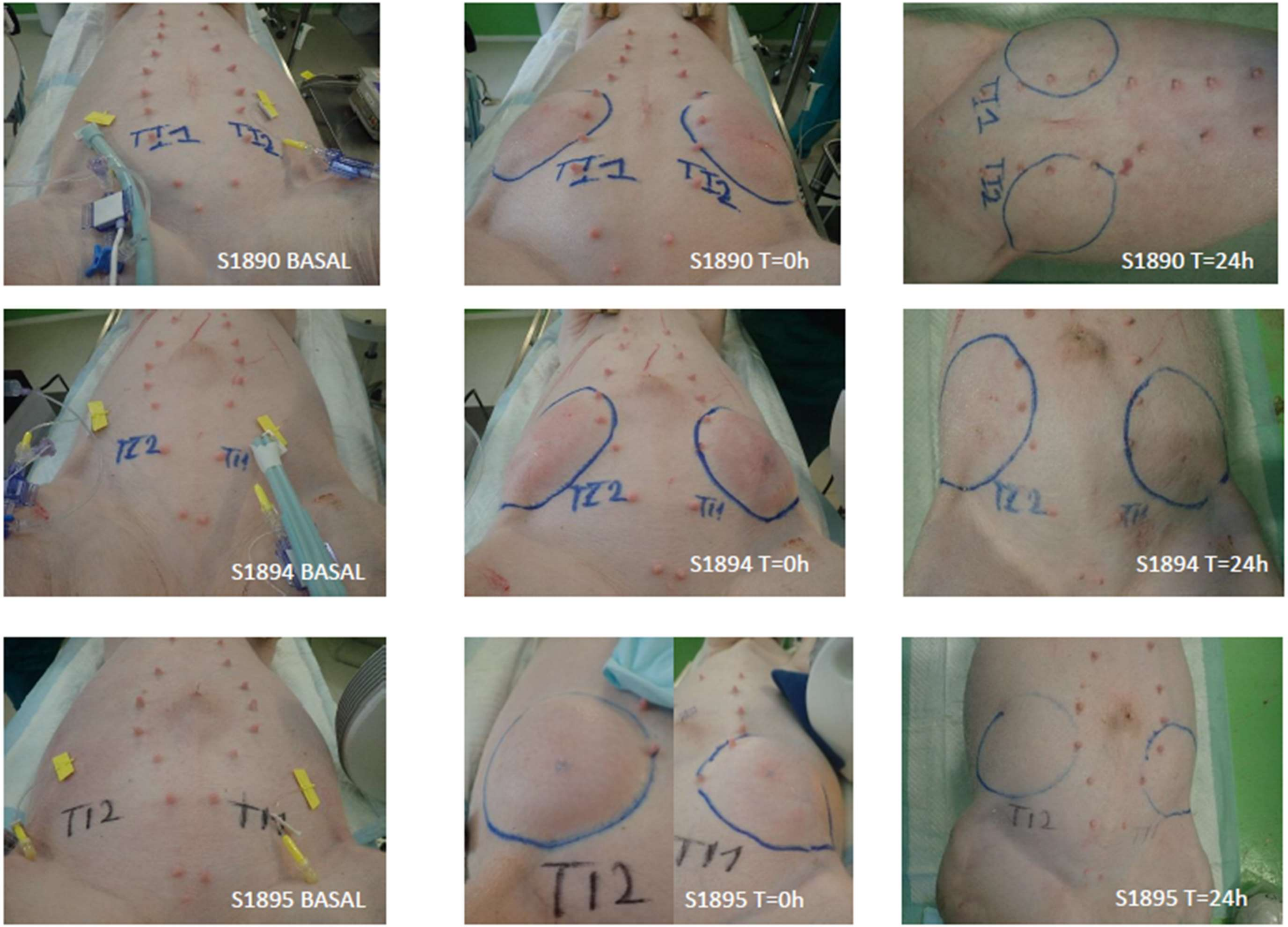

TI1 = warmed fIGSC 20%; TI2 = non-warmed fIGSC 20%. In animal S1895, TI2 was not considered for analysis.

Suppl. Figure 9: Histopathology Assessment of Local Tolerability of 160 mL warmed and non-warmed fIGSC 20% in Pigs

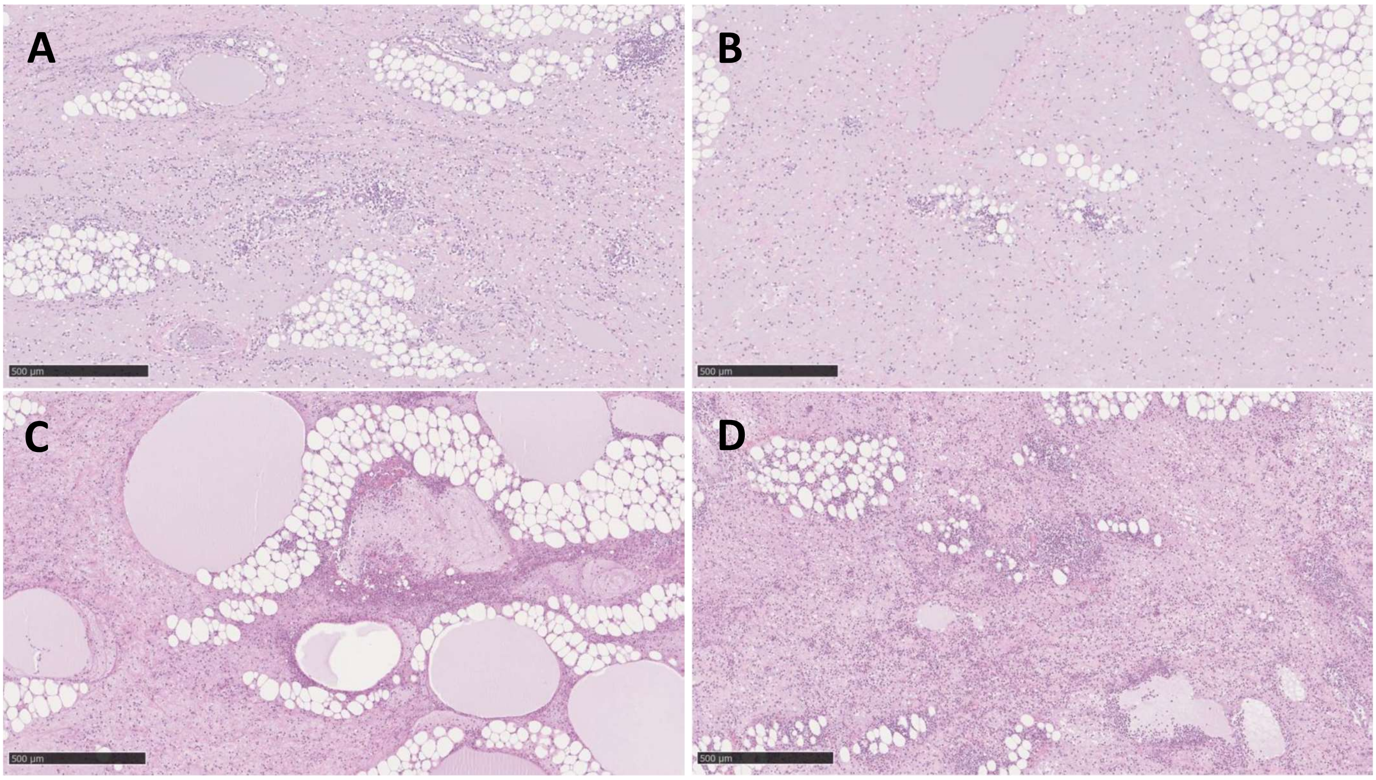

A. Sample No. S1890R; 50 mL warmed fIGSC 20% (2 mL/min); inflammation of subcutaneous tissue (Grade 2), edema of subcutaneous tissue (Grade 2); H&E, scale bar 500 µm. **B.** Sample no. S1894L; 50 mL warmed fIGSC 20% (2 mL/min); inflammation of subcutaneous tissue (Grade 1), edema of subcutaneous tissue (Grade 3); H&E, scale bar 500 µm. **C.** Sample no. S1895L 50 mL warmed fIGSC 20% (2 mL/min); inflammation of subcutaneous tissue (Grade 2), edema of subcutaneous tissue (Grade 3); H&E, scale bar 500 µm. **D.** Sample no. S1890L 50 mL non-warmed fIGSC 20% (2 mL/min); inflammation of subcutaneous tissue (Grade 3), edema of subcutaneous tissue (Grade 3); H&E, scale bar 500 µm.
